# Queries in Medicine and Natural History

**Published:** 1812-05

**Authors:** 


*To the Editors of the Medical and Physical Journal.*

GENTLEMEN,

HAVING from time to time observed QUERIES in your Journal, which have occasionally been very satisfactorily answered, I have ventured to request of your ingenious and learned correspondents, a solution of, or observations on, the following. In a hope that you will indulge me with their insertion, and that some of the numerous correspondents of the Medical Journal will favor your readers with remarks on them, and which I beg to say will be peculiarly gratifying to myself. I remain, &c. A. B.

*Liverpool, March* 2, 1812.

1. Among the species of the genus *Euphorbium*, natives of England, are there any that have been ascertained to possess medicinal properties? They are all of them powerful, and perhaps acrid, stimulants; and one of them has been employed with success in icterus, on the continent, it is asserted in the *Bibliotheque Germanique*.

2. Are there any authentic facts proving the curative properties of the *Agaricus deliciosus* and the *A. piperitus* in pulmonary consumption? Are there on record any facts to prove the use of the *Ranunculus aquaticus* in the above complaint: or has it been noticed at all as a remedy in pulmonic diseases, since, in the first volume of this Journal, it was stated to be employed with success in consumption, by an empiric?

3. What birds visit the fens of Cambridgeshire and Lincolnshire in the summer, and what are those which appear there in the winter? Does the *Ardea grus* ever now visit these flat regions? Perceiving that you sometimes, and as your title authorises, touch on natural history, I have ventured to put these two questions. Allow me also to ask if there is any arranged catalogue, or catalogue raisonnée, of Mr. Bullock's Museum? A collection so extensive cannot be understood without such a companion.

4. Is there any authentic history of the tree that produces the Caoutchoc, or elastic gum, with the mode in which the Indians prepare that singular substance?

